# Effects of Dietary Terpinen-4-ol on Oxidative Stress and Mitochondrial Biogenesis in the Liver of Broilers with Pulmonary Hypertension Syndrome

**DOI:** 10.3390/ijms26167702

**Published:** 2025-08-09

**Authors:** Xinyue Jiang, Liang Fei, Yayun Yang, Jiao Han, Zhaoxin Tang, Jianzhao Liao, Lianmei Hu, Ying Li, Jiaqiang Pan

**Affiliations:** College of Veterinary Medicine, South China Agricultural University, Guangzhou 510642, China; 20222029009@stu.scau.edu.cn (X.J.); feiliang@stu.scau.edu.cn (L.F.); 965875913@stu.scau.edu.cn (Y.Y.); 20243073031@stu.scau.edu.cn (J.H.); tangzx@scau.edu.cn (Z.T.); liaojz@scau.edu.cn (J.L.); hulianmei@scau.edu.cn (L.H.); lying@scau.edu.cn (Y.L.)

**Keywords:** broiler, liver, pulmonary hypertension syndrome, oxidative stress, mitochondrial biogenesis

## Abstract

Pulmonary hypertension syndrome (PHS), a metabolic disorder causing economic losses in broilers, arises from hypoxia-induced portal hypertension and liver cirrhosis, triggering mitochondrial oxidative damage, excessive ROS production, and altered mitochondrial biogenesis. This study explored terpinen-4-ol (T4O), known for antimicrobial and anti-inflammatory properties, in mitigating PHS. Broilers were divided into four groups, including PHS-affected birds with/without T4O supplementation. Analyses revealed that PHS birds exhibited reduced antioxidant capacity, elevated MDA and ROS levels, increased mitochondrial numbers, and upregulated expression of oxidative stress markers (Keap1, HO-1, Nrf-2) and mitochondrial biogenesis regulators (PGC-1α, Nrf-1, Tfam). T4O administration enhanced antioxidant activity, reduced ROS and MDA, suppressed compensatory mitochondrial proliferation, and downregulated Keap1/Nrf-2 and mitochondrial biogenesis pathways. These effects suggest that T4O alleviates hypoxia-driven oxidative stress and mitochondrial dysfunction in broilers. Findings highlight T4O’s potential as a therapeutic agent to mitigate PHS-related losses in poultry production.

## 1. Introduction

Ascites syndrome (AS), also known as pulmonary hypertension syndrome (PHS), is classified as a metabolic disease that predominantly affects young chickens. The disease has a significant impact on the broiler industry, causing a global economic loss of approximately 100 million dollars per year [1]. Clinically, PHS is characterized by the excessive accumulation of fluid within the body cavity, pulmonary congestion and edema, right ventricular dilation, and hepatic lesions. A multitude of factors contribute to the onset of this disease, including internal factors such as genetic factors associated with different strains and anatomical characteristics of avian lungs and external factors, including high-altitude conditions, cold and low-oxygen environments, inadequate ventilation, respiratory diseases, high-energy and high-protein diets, and excessive sodium intake. Among these factors, hypoxia is considered the most critical, as it leads to increased pulmonary arterial pressure, right ventricular failure, and elevated blood pressure within the hepatic portal circulation and abdominal visceral organs [2]. Currently, the specific mechanisms underlying this disease and the development of targeted pharmacological interventions remain hot topics of ongoing research.

Mitochondria are the primary energy source for cellular activities. The homeostasis of healthy cells is regulated by the quality and function of mitochondria [3]. The liver is one of the most metabolically active organs, which is significantly affected during the pathogenesis of PHS. Mitochondrial biogenesis refers to the growth and division of pre-existing mitochondria. Mitochondrial biogenesis is influenced by various environmental stressors, such as exercise, caloric restriction, low temperature, oxidative stress, cell division, renewal, and differentiation. This process is characterized not only by variations in the number of mitochondria but also by changes in their size and mass [4]. The enhanced mitochondrial biogenesis increases the copy number of mtDNA and protein subunits of metabolic enzymes, ultimately resulting in a greater metabolic capacity. Peroxisome proliferator-activated receptor gamma coactivator 1-alpha (PGC-1α) is a major regulator of mitochondrial biogenesis [5]. The activation of the PGC-1α pathway, which can occur through phosphorylation or deacetylation, initiates a cascade of nuclear transcription factors, including nuclear respiratory factor-1 (Nrf-1), Nrf-2, and estrogen-related receptor-α (ERR-α), culminating in the activation of the final effector, Tfam, which is involved in mtDNA transcription and replication. Activation of the PGC-1α pathway regulates mitochondrial oxidative phosphorylation and mitochondrial biogenesis, thereby mitigating oxidative damage to mitochondria and cells [6].

Oxidative stress induces cellular damage, which contributes to the aging process and gradual onset of apoptosis [7]. This phenomenon also activates pro-inflammatory factors that elicit inflammatory responses. As a result, these factors adversely affect the production performance of livestock and poultry, as well as the quality of animal products [8]. The regulatory network comprising multiple signaling pathways is interconnected and plays a critical role in regulating and maintaining oxidative balance homeostasis. Investigating the regulatory mechanisms of oxidative stress signaling pathways holds significant theoretical importance for the development of targeted regulators, the mitigation of oxidative stress, and the enhancement of production performance in animal husbandry.

Nuclear factor-E2-related factor 2 (Nrf-2) serves as a critical transcription factor within the antioxidant system, playing a fundamental role in the maintenance of redox balance. Under normal conditions, Nrf-2 binds to Keap1 in the cytoplasm, leading to Keap1-mediated ubiquitination and subsequent lysosomal degradation [9]. However, in response to exogenous or endogenous stimuli, Nrf-2 dissociates from Keap1, becomes liberated, and translocates to the nucleus. In the nucleus, Nrf-2 binds to Antioxidant Response Elements (AREs) and initiates the expression of antioxidant enzymes, including Heme Oxygenase-1 (HO-1), NQO-1, and GPX4 [10].

Terpinen-4-ol (T4O) is a monoterpene compound that constitutes a significant component of essential oils derived from various plant species. Research has demonstrated that this compound exhibits antioxidant, antibacterial, anti-inflammatory, and antitumor properties [11,12,13]. T4O exerts multifaceted bioactivities through distinct mechanisms: its antifungal action involves binding to ergosterol in fungal cell walls to form transmembrane channels that increase membrane permeability, ultimately causing cell death [11]; as an insecticidal agent, it targets Na^+^/K^+^-ATPase in neural tissues, disrupting ionic balance and neural transmission to induce insect mortality; in anticancer applications, it induces programmed cell death via modulation of apoptosis-related proteins, particularly through the Bax/Bcl-2 pathway; meanwhile, in antioxidant and anti-inflammatory contexts, T4O synergizes with α-terpinene and chlorogenic alcohol to demonstrate moderate NO radical-scavenging activity and reducing power, potentially mitigating oxidative stress through the regulation of antioxidant proteins like human catalase 5 [14]. Therefore, T4O is applicable to fields such as medicine, consumer products, and agricultural hygiene, with a wide range of application scenarios and a broad scope. We hypothesize that, as a natural antioxidant, the incorporation of an appropriate concentration of terpinen-4-ol into the diet may effectively reduce the production of free radicals in the body, enhance antioxidant capacity, and alleviate oxidative stress associated with mitochondrial biogenesis during PHS.

## 2. Results

### 2.1. Pathological Changes in Different Group

**Figure 1:**
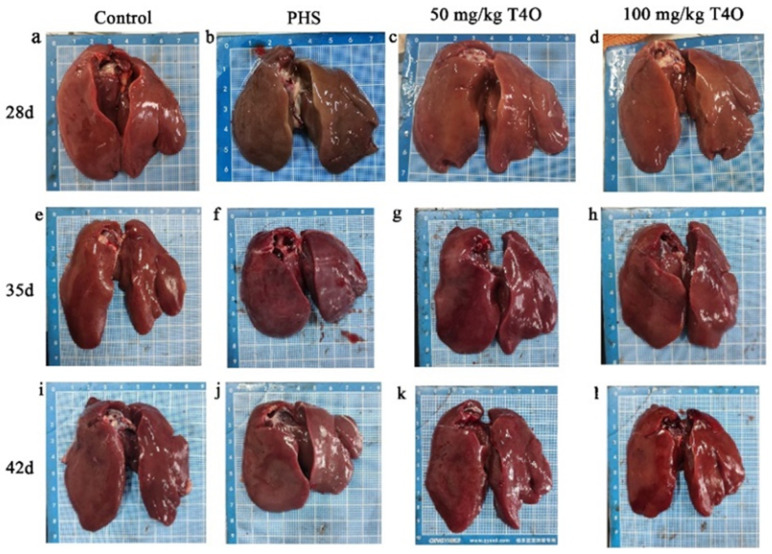
Pathological changes in the liver from four groups. Rows represent different groups; columns represent different ages. (**a**) The liver of the 28-day-old control group. (**b**) The liver of the 28-day-old PHS group. (**c**) The liver of the 28-day-old 50 mg/kg T4O group. (**d**) The liver of the 28-day-old 100 mg/kg T4O group. (**e**) The liver of the 35-day-old control group. (**f**) The liver of the 35-day-old PHS group. (**g**) The liver of the 35-day-old 50 mg/kg T4O group. (**h**) The liver of the 35-day-old 100 mg/kg T4O group. (**i**) The liver of the 42-day-old control group. (**j**) The liver of the 42-day-old PHS group. (**k**) The liver of the 42-day-old 50 mg/kg T4O group. (**l**) The liver of the 42-day-old 100 mg/kg T4O group.

**Figure 2:**
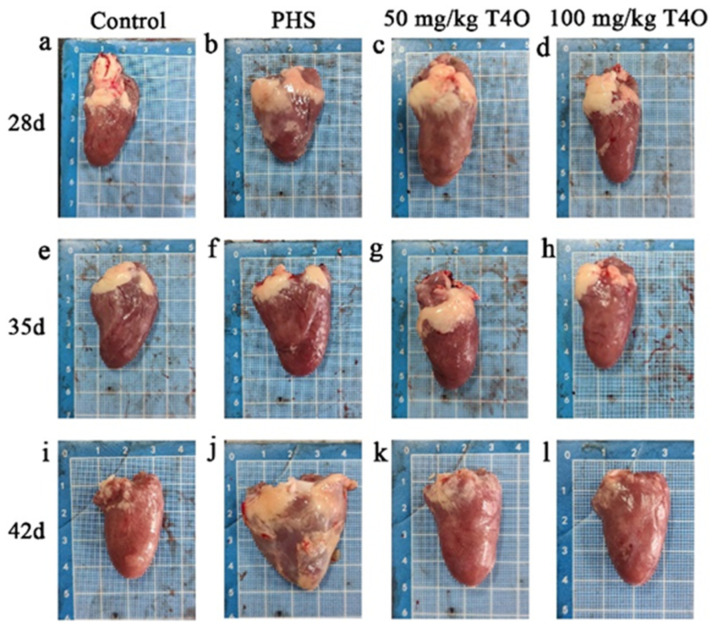
Pathological changes in the heart from four groups. Rows represent different groups; columns represent different ages. (**a**) The heart of the 28-day-old control group. (**b**) The heart of the 28-day-old PHS group. (**c**) The heart of the 28-day-old 50 mg/kg T4O group. (**d**) The heart of the 28-day-old 100 mg/kg T4O group. (**e**) The heart of the 35-day-old control group. (**f**) The heart of the 35-day-old PHS group. (**g**) The heart of the 35-day-old 50 mg/kg T4O group. (**h**) The heart of the 35-day-old 100 mg/kg T4O group. (**i**) The heart of the 42-day-old control group. (**j**) The heart of the 42-day-old PHS group. (**k**) The heart of the 42-day-old 50 mg/kg T4O group. (**l**) The heart of the 42-day-old 100 mg/kg T4O group.

The necropsy results are shown in Figure 1 and Figure 2. In the control group, the heart and liver appeared normal in color, showing no pathological changes. In the PHS group, some broilers revealed liver atrophy, a darkened color, blunt and rounded edges, marked myocardial hypertrophy, and right ventricular dilation. In both T4O groups, there were some degrees of cardiac dilation and thickening; however, the liver appeared normal in color with no noticeable lesions.

### 2.2. Ascites Heart Index and Hepatic Coefficient

At all-time points, the AHI values of the PHS group were significantly elevated in comparison to the control group (*p* < 0.01). The AHI values in both T4O groups were significantly lower than those in the PHS group at all three time points (Table 1).

The hepatic coefficients of the PHS group at 28, 35, and 42 days of age were significantly higher than those in the control group (*p* < 0.05, *p* < 0.01, *p* < 0.05). At 35 and 42 days of age, the hepatic coefficients in both T4O groups were significantly reduced compared to the PHS group (Table 1).

### 2.3. Effects of T4O on Hepatic Enzymes Activity of PHS-Affected Broilers

On day 28 and day 42, the activity of AST in the PHS group was significantly higher than that in the control group (*p* < 0.05, *p* < 0.01, respectively). At each time point, the activity of AST in the 50 mg/kg T4O group was significantly lower than that in the PHS group. Throughout all time points, the activity of ALT in the PHS group was significantly elevated compared to the control group, while the activity of ALT in the 50 mg/kg T4O group was significantly lower than that in the PHS group (Table 2).

### 2.4. Effects of T4O on Hepatic Histopathological Changes in PHS-Affected Broilers

**Figure 3:**
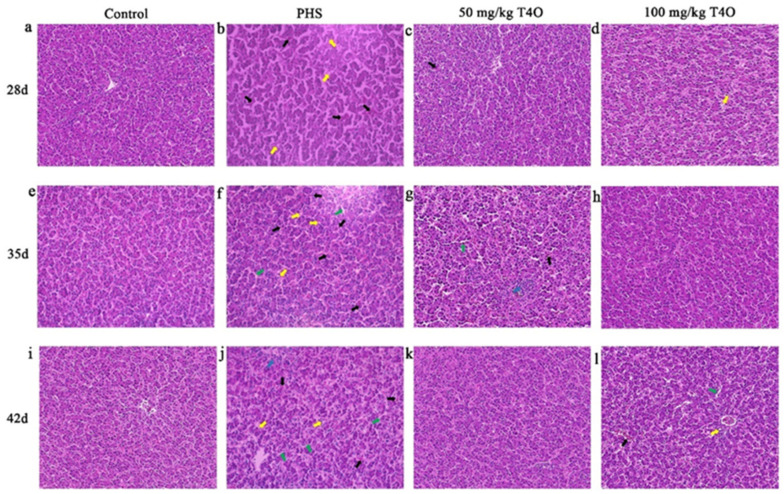
(**a**–**l**) Histopathological changes in the liver of four groups (HE staining, 400×). Rows represent different groups; columns represent different ages. The yellow arrows indicate vacuolar fatty degeneration of hepatocytes, the green arrows indicate sinusoidal dilation, the blue arrows indicate inflammatory cell infiltration, and the black arrows represent sinusoidal congestion.

The HE staining results showed that the hepatocytes in the control group were arranged in an orderly manner, with clear boundaries between the hepatic cords and sinusoids, and no significant pathological changes were observed in the intercellular spaces. In the PHS group at all stages, abnormal dilation of the hepatic sinusoids was observed, accompanied by significant congestion forming the hepatic blood sinusoids, and noticeable vacuolation was also observed. Additionally, inflammatory cell infiltration was present in the PHS group on day 42. In the 50 mg/kg T4O group on day 28, slight congestion in the hepatic sinusoids and the neatly arranged hepatic cords were observed. In the 100 mg/kg T4O group, occasional cells exhibited vacuolization. On day 35, the 50 mg/kg T4O group showed mild sinusoidal dilation and congestion, with minimal inflammatory cell infiltration. On day 42, the 100 mg/kg T4O group exhibited clear boundaries of hepatic cords, partial dilation of the hepatic sinusoids, and cytoplasmic coagulative eosinophilia and vacuolization (Figure 3).

### 2.5. Effects of T4O on Hepatic Mitochondria Ultrastructure of PHS-Affected Broilers

**Figure 4:**
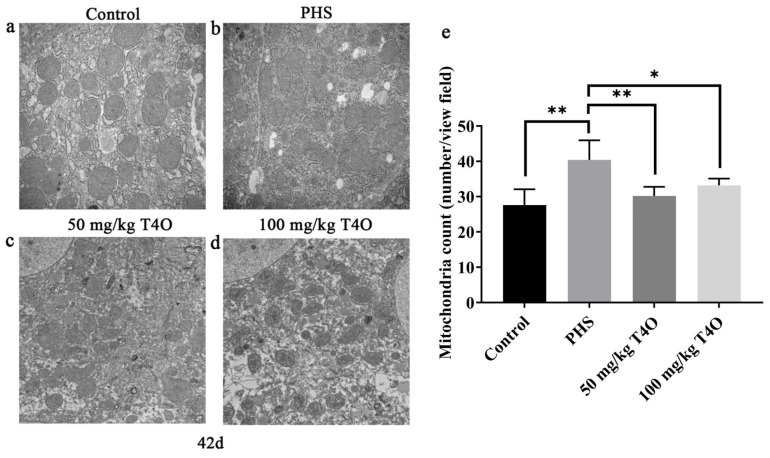
Transmission electron microscopy photograph changes in the liver mitochondria from the control group (**a**), PHS group (**b**), 50 mg/kg T4O group (**c**), and 100 mg/kg T4O group (**d**) on day 42 (20,000×). Number of mitochondria in the liver of 42-day broilers (**e**). * *p* < 0.05, ** *p* < 0.01.

In the control group, the mitochondria exhibited a clear morphology, characterized by orderly arranged cristae and regular mitochondrial shapes (Figure 4a). In contrast, the mitochondria in the PHS group varied in size, with some cristae showing dissolution and some mitochondria displaying distorted shapes (Figure 4b). In both T4O groups, the mitochondria maintained clear morphology (Figure 4c,d). In addition, the number of mitochondria increased in the PHS group, while it decreased significantly in the T4O group compared with the PHS group (Figure 4e).

### 2.6. Effects of T4O on Oxidative Stress in Liver

**Figure 5:**
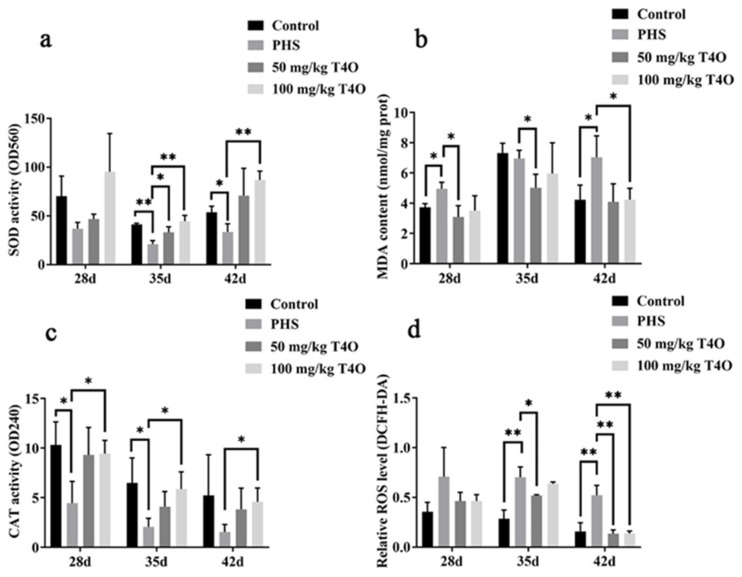
Effects of T4O on oxidative stress SOD (**a**), CAT (**b**), MDA (**c**), and ROS (**d**) of PHS-affected broilers. * *p* < 0.05, ** *p* < 0.01.

On day 28, there were no significant differences in ROS levels among the groups. However, on day 35 and day 42, the ROS levels in the PHS group were significantly higher than those in the control group (*p* < 0.01). Compared to the PHS group, the ROS levels in the 50 mg/kg T4O group were significantly reduced on day 35 (*p* < 0.05). On day 42, both T4O groups showed a significant downward trend in ROS levels when compared to the PHS group (*p* < 0.01) (Figure 5d).

On day 35, the PHS group exhibited reduced activities of SOD and CAT and elevated levels of MDA compared with the control group. T4O (50 mg/kg and 100 mg/kg) treatments improved SOD and CAT activities and reduced MDA levels, especially on day 35 and day 42 (Figure 5a-c).

**Figure 6:**
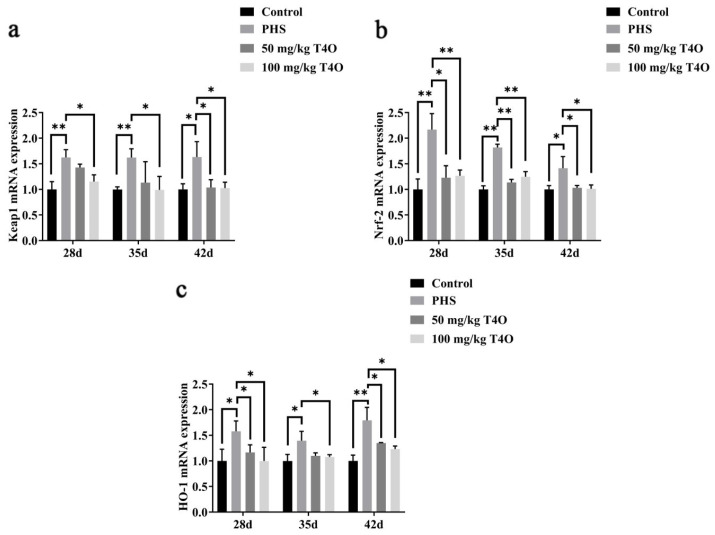
Effect of T4O on the expression of genes related to oxidative stress: (**a**) the mRNA expressions of Keap1; (**b**) the mRNA expressions of Nrf-2; (**c**) the mRNA expressions of HO-1. * *p* < 0.05, ** *p* < 0.01.

On day 28 and day 42, the mRNA expressions of HO-1, Nrf-2, and Keap1 were higher in the PHS group than in the control group. T4O treatment, especially at 100 mg/kg dosage, significantly reduced the expression of these markers compared to the PHS group. The protein expression showed a similar trend (Figure 6).

### 2.7. Effects of T4O on Mitochondrial Biogenesis in Liver

**Figure 7:**
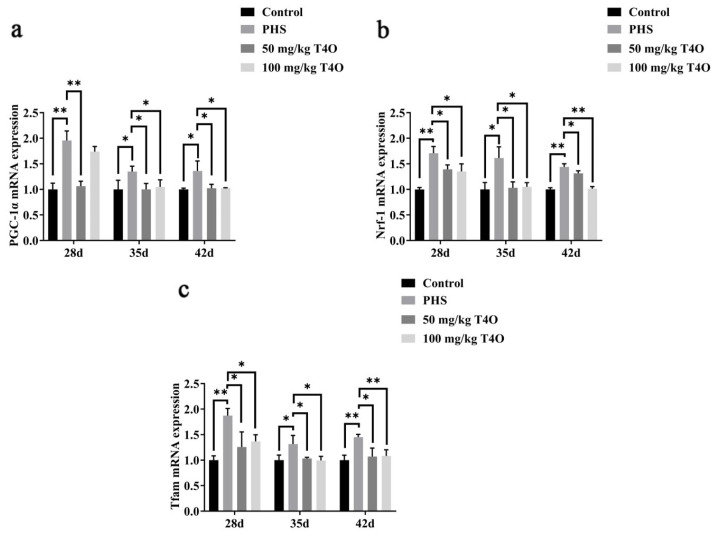
Effect of T4O on the expression of genes related to mitochondrial biogenesis. Note: (**a**) the mRNA expressions of PGC-1α; (**b**) the mRNA expressions of Nrf-1; (**c**) the mRNA expressions of Tfam. * *p* < 0.05, ** *p* < 0.01.

On day 28, day 35, and day 42, the PHS group showed significantly higher expression of PGC-1α, Nrf-1, and Tfam mRNA compared to the control group. T4O treatment (50 mg/kg and 100 mg/kg) significantly reduced the expression of these markers, with a more pronounced effect observed at the higher dosage, especially for Nrf-1 and Tfam (Figure 7).

## 3. Discussion

This study aims to investigate the effects of T4O on oxidative stress and mitochondrial biogenesis in the livers of broilers with pulmonary hypertension syndrome. The widely accepted theory regarding the pathogenesis of PHS is based on the pulmonary hypertension hypothesis, which posits a sequence of events: inducing factors → hypoxia → pulmonary hypertension → right ventricular hypertrophy → right heart failure → liver congestion → ascites formation [15]. The liver, one of the primary organs impacted by PHS, plays a crucial role in the development of ascites. During PHS, venous return is obstructed, leading to increased capillary hydrostatic pressure, which subsequently leads to portal hypertension. This condition results in venous congestion and edema in abdominal organs, leading to liver swelling, and the leakage of tissue fluid from the liver into the abdominal cavity, forming ascites [15]. Additionally, tissue hypoxia itself can cause partial liver dysfunction. For example, extensive epidemiological studies have shown that high-altitude hypoxic environments induce the accumulation of oxygen free radicals in the body, potentially leading to hepatocyte membrane damage and liver injury, resulting in oxidative stress imbalance [16], with significantly reduced SOD activity and elevated MDA levels [17].

T4O is a monoterpene compound and is a primary component of essential oils found in various plant species [11]. This study confirmed that T4O relieved oxidative stress response during PHS in broilers. Some studies discovered that T4O can inhibit high-glucose-induced abnormal proliferation of vascular smooth muscle cells (VSMCs), reduce ROS levels, downregulate the expression of KLF4 in VSMCs induced by high glucose, and upregulate the expression of the antioxidant signals Nrf-2 and HO-1 [18]. These research results are consistent with the findings of our study. The Keap1/Nrf-2/HO-1 signaling axis is an indispensable pathway in the antioxidant stress response, where Nrf-2 serves as a crucial transcription factor that coordinates the antioxidant response, effectively eliminating ROS [9]. Additionally, Nrf-2 is also a key transcription factor in mitochondrial biogenesis, regulated by PGC1. This study confirmed that T4O has an inhibitory effect on PHS through the Keap1/Nrf-2/HO-1 signaling pathway.

In this study, broilers in the PHS group exhibited a significantly higher AHI and hepatic coefficient compared to the control group. Pulmonary hypertension caused right heart failure and subsequent blood stasis in the liver, as well as cirrhosis [19]. Additionally, weight loss contributed to an elevated liver coefficient. Blood biochemical analysis showed that the PHS group had higher AST and ALT levels, indicating liver damage [20]. HE staining observations revealed hepatic congestion, inflammation, and fibrosis in PHS broilers. These changes confirmed the liver damage caused by PHS [21]. Furthermore, on 35 and 42 days of age, the hepatic coefficients, the AST and ALT levels in the 50 mg/kg T4O group were significantly lower than those in the PHS group, and the pathological changes in liver tissue sections were markedly alleviated, demonstrating that T4O has a protective effect on liver damage.

When broilers develop ascites, their liver mitochondrial function becomes impaired, leading to a significant leakage of electrons from the respiratory chain. This results in reduced oxygen consumption for ATP production and increased oxygen consumption for free radical generation, ultimately leading to insufficient energy metabolism and oxidative stress. Improving the insufficient energy metabolism and oxidative stress in liver mitochondria can help reduce the occurrence of ascites [22]. To ensure a sufficient energy supply, cells generate new mitochondria through mitochondrial biogenesis, maintaining the mitochondrial population and preserving mitochondrial functional structure. In this study, transmission electron microscopy showed that, although the number of mitochondria increased in the PHS group, their size was reduced. The administration of T4O effectively reduced the changes in mitochondrial number and morphology. SOD and CAT are key antioxidant enzymes in animals that help counteract oxidative stress from external stimuli, and their activity is influenced by free radicals [23]. Both acute and chronic oxidative stress can significantly affect the activity of antioxidant enzymes and increase MDA levels. MDA is one of the primary end products of lipid peroxidation chain reactions. Its concentration indirectly reflects both the level of free radical generation and the extent of lipid peroxidation in tissues and cells [24]. In this study, the PHS group showed significantly reduced SOD and CAT activities, along with increased MDA and ROS levels, which is consistent with the occurrence of oxidative stress in the body. The T4O-treated groups were able to restore antioxidant enzyme levels to normal, reduce MDA content, and enhance the antioxidant capacity of the broilers during the rearing period [25]. Research results proved that T4O could serve as a potential oxidative stress mitigator.

To further validate our hypothesis, we conducted analyses of the expressions of relevant genes and proteins related to oxidative stress and mitochondrial biogenesis. The Keap1/Nrf-2 signaling pathway is a critical mechanism for defending against oxidative stress induced by external stimuli. It maintains redox homeostasis in the body and mitigates tissue and cellular damage caused by external stressors or endogenous free radicals and reactive oxygen species by regulating the activity of downstream enzymes or gene expression [26]. Mitochondrial biogenesis is primarily regulated by the transcriptional coactivator PGC-1 [27]. Once activated, PGC-1α translocates to the nucleus, where it activates Nrf-1/Nrf-2, leading to increased expression of Tfam [28]. Tfam, in turn, promotes the replication and transcription of mtDNA and the production of mitochondrial proteins, ultimately enhancing mitochondrial biogenesis [29]. The experimental results showed that, compared to the control group, the mRNA expression levels of Keap1, HO-1, PGC-1α, Nrf-1, Nrf-2, and Tfam were significantly increased in the liver of broilers in the PHS group. These findings suggest that, during hypoxia in the process of PHS, the expression of the Keap1/Nrf-2/HO-1 pathway is upregulated, leading to oxidative stress in the body. Concurrently, mitochondrial biogenesis is activated, with increased expression of the PGC-1α/Nrf-s/Tfam pathway, which results in an increase in mitochondrial numbers to produce more ATP [30]. This helps to mitigate the damage caused by excessive ROS production, enhancing the antioxidative and protective capabilities of the body and cells [31]. In this experiment, the administration of two different doses of T4O led to a reduction in the expression of relevant genes and proteins to varying degrees, and the ROS levels in the body also showed a significant decreasing trend. Therefore, we speculate that the reduction in the expression of active biogenesis-related factors in the liver tissue of PHS broilers may be due to T4O’s ability to directly or indirectly eliminate ROS and enhance the body’s antioxidant capacity. Excessive ROS leads to mtDNA damage and electron transport chain (ETC) dysfunction, resulting in newly generated mitochondria with potential functional defects [32]. If these defective mitochondria escape quality control (QC) and fail to be cleared, ROS leakage further escalates [33]. T4O may restore mitochondrial homeostasis and ameliorate energy metabolism disorders by suppressing this pathological process. At the same time, the inhibition of PGC-1α, Nrf-1, Nrf-2, Tfam, Keap1, and HO-1 expression likely contributes to the reduction in oxidative stress and mitochondrial biogenesis [34], thereby mitigating the liver damage caused by PHS and providing a certain therapeutic effect. Of interest, the hepatoprotective effect of T4O was comparable to that of other natural antioxidants (resveratrol, quercetin, etc.) in poultry production [35,36,37] and similarly significantly increased the antioxidant capacity against liver injury in broiler chickens, further demonstrating the therapeutic effectiveness of T4O. In addition, T4O has a simpler composition and a clearer mechanism of monomer compounds compared to known plant extracts. It is widely found in plant essential oils, with clearer application prospects and unique natural advantages. However, our study still has some limitations. Although mitochondrial biogenesis-related markers were tested in this study, key parameters used to assess mitochondrial function (ATP content, mitochondrial membrane potential, etc.) were not adequately measured. Furthermore, mitochondrial quality control is not only biogenesis-dependent but also involves the participation of mitochondrial autophagy and mitochondrial dynamics. Therefore, we will explore whether T4O is equally involved in the regulation of mitochondrial autophagy and mitochondrial dynamics-related regulation in future studies. We will also utilize the assay to quantify the improvement of mitochondrial function by T4O.

## 4. Materials and Methods

### 4.1. Broiler Feeding and Grouping

One-day-old Arbor Acres (AA) broilers were routinely immunized and raised under controlled conditions (20 ± 2 °C, 50 ± 5% humidity, 23 h light/1 h dark cycle). On day 21, a total of 144 broilers were randomly assigned to four groups: a control group, a PHS group, a low-dose group (50 mg T4O/kg BW per day), and a high-dose group (100 mg T4O/kg BW per day). T4O (Aladdin, Shanghai, China) was applied to the feed by SiO_2_ adsorption at a ratio of 1:1. From 21 days, control and PHS groups received standard feed, while T4O groups were fed T4O-mixed feed until 42 days. This work was approved by the Animal Ethics Committee of South China Agricultural University (Approval No: 2024F072, Approval Date: 4 March 2024).

### 4.2. PHS Model Establishment and Sampling

On the 21st day of age, broilers in the PHS and T4O groups received a 0.35 mL injection of CM-32 ion-exchange cellulose particle suspension via the wing vein [19], containing 0.02 g cellulose particles (MCE, South Brunswick Township, NJ, USA) and 150 U heparin sodium (Biosharp, Hefei, China) per milliliter. The control group received saline. This animal modeling methodology references our previous study [19]. On 28, 35, and 42 days of age, six broilers per group were dissected, and blood, serum, heart, and liver samples were collected.

### 4.3. Ascites Heart Index

The heart was collected, the atria and large vessels were removed, and any blood clots within the heart chambers were cleared before weighing the total ventricular weight (TV). The heart was then dissected along the margin of the interventricular septum to measure the right ventricular weight (RV). The Ascites Heart Index (AHI) for each chicken was calculated as the RV/TV ratio.

### 4.4. Hepatic Coefficient Evaluations

The liver was collected, and attached tissues such as adipose tissue were removed. The surface blood was rinsed with pre-cooled PBS buffer, and the liver was weighed. The body weight and liver weight of each sample were recorded. Equation (1) is as follows:

Hepatic coefficient = (Liver weight (g) ∗ 100%)/body weight (g)(1)

### 4.5. Serum Biochemical Analysis of Hepatic Enzymes

Serum samples collected via the wing vein were analyzed using a fully automated biochemical analyzer (BS-380, Mindray, Shenzhen, China). The parameters included ALT and AST. Calibration and quality control were conducted. Follow the standard procedure of ‘calibration–quality control–testing’, strictly adhering to the manufacturer’s reagent kit instructions to confirm whether the quality control samples are within control limits, and finally export the valid data (Mindray, Shenzhen, China).

### 4.6. Histopathological Examination of Liver

Liver tissue fixed in 4% paraformaldehyde was dehydrated, embedded, sectioned, stained with HE (hematoxylin and eosin), and mounted; the pathological changes in liver tissue were observed under an optical microscope (DM1000, Leica, Wetzlar, Germany); and the shooting parameters of different samples were kept consistent. Liver histopathology was assessed in ≥ 5 random fields per H&E-stained section (400× magnification). Key readouts included the following: (1) vacuolar fatty degeneration, (2) sinusoidal dilation, (3) inflammatory cell infiltration, and (4) sinusoidal congestion.

### 4.7. Mitochondrial Ultrastructure Observation of Liver

Liver tissue (1 mm^3^) was fixed in 2.5% glutaraldehyde, dehydrated, and polymerized for embedding. Ultrathin sections were stained with citrate lead and uranyl acetate and then observed under a transmission electron microscope (H-7500, Hitachi, Tokyo, Japan). Under the same multiple, three fields of view were observed in each sample to record the number of mitochondria, and the average value was calculated as the representative value.

### 4.8. Detection of Oxidative Stress in Liver

Liver tissue was homogenized, and the supernatant was collected. The concentrations of malondialdehyde (MDA) and the activities of superoxide dismutase (SOD) and catalase (CAT) were quantified using assay kits (Solarbio, Beijing, China). Fresh liver tissue was collected and minced, followed by digestion to isolate cells. The cells were incubated with the fluorescent probe DCFH-DA (MCE, NJ, USA) at 37 °C for 20–30 min. ROS was then detected using a flow cytometer (CYTOFLEX, Beckman, Brea, CA, USA).

### 4.9. Gene Expression Analysis by Real-Time Quantitative PCR

Liver tissue was used for total RNA extraction with Trizol reagent (AG, Changsha, China). The HiScript II Q RT SuperMix for qPCR kit (Vazyme, Nanjing, China) was used to reverse transcribe the RNA into cDNA. Real-time quantitative PCR performed with the ChamQ SYBR Color qPCR Master Mix kit (Vazyme, Nanjing, China) and the analytikjena qTOWER3G (Analytik Jena, Jena, Germany). Gene sequences were obtained from NCBI GenBank, with β-actin as the reference gene. Primers, designed using Primer Premier 5 and synthesized by Sangon Biotech (Shanghai, China) Co., Ltd., are listed in Table 3.

### 4.10. Statistical Analysis

Quantitative data were expressed as mean ± SD. Data were analyzed using one-way ANOVA and multiple t-tests. All data were statistically analyzed using GraphPad Prism 7.0 (GraphPad Inc., La Jolla, CA, USA).

## 5. Conclusions

In conclusion, the results indicated that PHS can promote hepatic mitochondrial biogenesis, likely due to a compensatory response mechanism. T4O was found to improve liver damage in PHS broilers and demonstrated a definite therapeutic effect by alleviating oxidative stress in liver tissue and mitigating the compensatory response of mitochondrial biogenesis. The effects did not exhibit dose-dependency. However, considering T4O’s distinctive odor and the goal of maximizing economic benefits, a dose of 50 mg/kg T4O is recommended as the clinical additive dosage, or further research could be conducted to determine the optimal additive dose.

## Figures and Tables

**Table 1:** Ascites heart index (RV/TV) and hepatic coefficient for each group.

Groups		Age		
		28 d	35 d	42 d
AHI (g/g)	Control	0.211 ± 0.023	0.213 ± 0.006	0.214 ± 0.022
	PHS	0.280 ± 0.030 ^A^	0.308 ± 0.021 ^A^	0.310 ± 0.055 ^A^
	50 mg/kg T4O	0.230 ± 0.026 ^b^	0.232 ± 0.015 ^B^	0.236 ± 0.019 ^b^
	100 mg/kg T4O	0.237 ± 0.022 ^b^	0.225 ± 0.014 ^B^	0.225 ± 0.020 ^B^
Hepatic coefficient (g/g)	Control	0.022 ± 0.001	0.021 ± 0.001	0.020 ± 0.002
	PHS	0.027 ± 0.003 ^a^	0.025 ± 0.002 ^A^	0.028 ± 0.008 ^a^
	50 mg/kg T4O	0.025 ± 0.002	0.022 ± 0.001 ^b^	0.020 ± 0.001 ^b^
	100 mg/kg T4O	0.024 ± 0.003	0.021 ± 0.001 ^B^	0.020 ± 0.002 ^b^

^A^ *p* < 0.01 vs. Control; ^a^ *p* < 0.05 vs. Control; ^B^ *p* < 0.01 vs. PHS; and ^b^ *p* < 0.05 vs. PHS.

**Table 2:** Serum AST/ALT activity (U/L) for each group.

Groups		Age		
		28 d	35 d	42 d
AST	Control	318.40 ± 18.54	212.20 ± 9.72	273.05 ± 51.85
	PHS	435.83 ± 51.32 ^a^	384.73 ± 17.35 ^A^	345.70 ± 58.28
	50 mg/kg T4O	291.67 ± 4.01 ^B^	299.28 ± 36.00 ^B^	255.00 ± 13.95 ^b^
	100 mg/kg T4O	288.43 ± 3.82 ^B^	327.58 ± 65.34	301.18 ± 20.20
ALT	Control	4.48 ± 0.69	5.28 ± 0.60	5.23 ± 0.91
	PHS	6.55 ± 0.31 ^A^	7.56 ± 0.82 ^A^	6.75 ± 0.55 ^a^
	50 mg/kg T4O	5.08 ± 0.56 ^B^	6.18 ± 0.76 ^b^	5.43 ± 0.45 ^B^
	100 mg/kg T4O	5.05 ± 1.31	6.22 ± 0.73 ^b^	6.58 ± 0.21

^A^ *p* < 0.01 vs. Control; ^a^ *p* < 0.05 vs. Control; ^B^ *p* < 0.01 vs. PHS; and ^b^ *p* < 0.05 vs. PHS.

**Table 3:** Real-time quantitative PCR primer sequences.

Primer Name	Primer Sequence (5′ to 3′)
β-Actin-F	GTTGGTATGGGCCAGAAAGA
β-Actin-R	CCGTGTTCAATGGGGTACTT
PGC-1α-F	GCCAAACAAAGGGAGAGAC
PGC-1α-R	CACCAAAAACTTCAAACCG
Nrf-2-F	ACTGGGGGTGGAAGTGATGC
Nrf-2-R	GCTCTCCCTGTGCTGTGCTG
Keap1-F	TCAACTGGGTGCAGTACGAC
Keap1-R	TCTGCGCCAGGTAATCCTTG
Nrf-1-F	CTGTGTCCCTCATCCAGGTT
Nrf-1-R	CCAGTTCTGCTCCACCTCTC
HO-1-F	GCTGGGAAGGAGAGTGAGAGGAC
HO-1-R	GCGACTGTGGTGGCGATGAAG
Tfam-F	TTCTCAAAAGCAGCCATAC
Tfam-R	TTCACGTCCAAGTTCAACC

## Data Availability

Data is contained within this article.

## Abbreviations

The following abbreviations are used in this manuscript:

AA	Arbor Acres
AHI	Ascites heart index
ALT	Alanine aminotransferase
AS	Ascites syndrome
AST	Aspartate aminotransferase
ARE	Antioxidant response elements
BCA	Bicinchoninic acid
CAT	Catalase
DCFH-DA	2′,7′-Dichlorodihydrofluorescein diacetate
ERR-α	Estrogen-related receptor-α
GPX4	Glutathione peroxidase 4
HE	Hematoxylin and eosin
HO-1	Heme oxygenase-1
Keap1	Kelch-like ECH-associated protein 1
mtDNA	Mitochondrial deoxyribonucleic acid
MDA	Malondialdehyde
Nrf-1	Nuclear respiratory factor 1
Nrf-2	Nuclear respiratory factor 2
NQO-1	NAD(P)H quinone dehydrogenase 1
PGC-1α	Peroxisome proliferator-activated receptor-γ coactivator-1α
PHS	Pulmonary hypertension syndrome
ROS	Reactive oxygen species
RT-qPCR	Real-time fluorescence quantitative PCR
TFAM	Mitochondrial transcription factor A
SOD	Superoxide dismutase
